# MATHEMATICAL MODELLING APPROACH OF THE STUDY OF EBOLA VIRUS DISEASE TRANSMISSION DYNAMICS IN A DEVELOPING COUNTRY

**DOI:** 10.21010/Ajidv17i1.2

**Published:** 2022-12-22

**Authors:** MBAH, Godwin Christopher E., ONAH, Ifeanyi Sunday, AHMAN, Queeneth Ojoma, COLLINS, Obiora C., ASOGWA, Christopher C., OKOYE, Chukwudi

**Affiliations:** 1 Department of Mathematics, University of Nigeria, Nsukka, Nigeria; 2 Department of Mathematical Sciences, Confluence University of Science and Technology, Kogi State, Nigeria

**Keywords:** Mathematical models, Ebola, Stability analysis, Lyapunov function, Reproduction number, Numerical simulation

## Abstract

**Background:**

Ebola Virus causes disease both in human and non-human primates especially in developing countries. In 2014 during its outbreak, it led to majority of deaths especially in some impoverished area of West Africa and its effect is still witnessed up till date.

**Materials and Methods:**

We studied the spread of Ebola virus and obtained a system of equations comprising of eighteen equations which completely described the transmission of Ebola Virus in a population where control measures were incorporated and a major source of contacting the disease which is the traditional washing of dead bodies was also incorporated. We investigated the local stability of the disease-free equilibrium using the Jacobian Matrix approach and the disease- endemic stability using the center manifold theorem. We also investigated the global stability of the equilibrium points using the LaSalle’s Invariant principle.

**Results:**

The result showed that the disease-free and endemic equilibrium where both local and globally stable and that the system exhibits a forward bifurcation.

**Conclusions:**

Numerical simulations were carried out and our graphs show that vaccine and condom use is best for susceptible population, quarantine is best for exposed population, isolation is best for infectious population and proper burial of the diseased dead is the best to avoid further disease spread in the population and have quicker and better recovery.

## Introduction

Ebola disease also known as Ebola hemorrhagic fever is a rare and deadly disease caused by infection with one of the Ebola virus species. Ebola Virus causes disease both in human and non-human primates. The Ebola virus have five identified species namely, Zaire Ebola virus (EBOV), Sudan Ebola virus (SUDV), Bundibugyo Ebola virus (BDBV), Reston Ebola virus (RESTV) and Tai Forest (Cote D’ivore) Ebola virus (TAFV). Four of these viruses (except RESTV) are known to cause Ebola virus disease in humans. In 1976, Ebola virus was first discovered near the Ebola River in what is now known as Democratic Republic of Congo, (Khan et al., 1999). Since then, there have been several outbreaks in Africa.

Human beings can get Ebola virus through direct contact (through broken skin or mucus membrane in the eyes, nose or mouth) with blood or body fluids of a person who is sick with Ebola disease or a person who has died from Ebola disease. Ebola virus disease (EVD) is a disease caused by Ebola virus which belongs to the Filoviridae virus family, transmitted by the handling of bush meats, contact with infected bats and from person- to- person via direct contact with infected bodily fluid, secretions, blood, contaminated environment/surfaces and organs, (Agusto *et al.*, 2017), (Saeed *et al.*, 2015), (WHO; 2016). One can equally get the disease through objects (such as syringes and needles) that have been contaminated with the virus from a person sick with Ebola disease or the body of a person who died from Ebola disease (CDC; 2016). The 2014 Ebola disease outbreak in West Africa is related with EBOV virus. This outbreak is the largest one in the history of the disease with multiple countries affected (WHO Ebola Team Response,2014). Rivers *et al.*, (2014) in their work stated that the outbreak began in Guinea on March 23, 2014. The outbreak led to wild spread and intense transmission in west African countries like Guinea, Liberia and Sierra Leone, as well as slight cases in five other countries namely, Nigeria, Senegal, Mali, Spain and USA, (Marisa *et al.*, 2014). This outbreak is considered the deadliest because it recorded 28,608 suspected cases and 11,306 deaths, (WHO, 2016). From this, one can see that the fatality rate, (0. 3952), of the disease is very high and thus needed urgent scientific and research attention.

In general, this viral disease has high case fatality rate which in past outbreaks varied between 25% and 90%, with an average of about 50%. However, it is known that the Zaire species, that was responsible for the West African outbreak, carries a higher death or fatality rate, Ahman *et al*. (2020).

Majority of deaths during the 2014 outbreak of Ebola virus occurred in the most impoverished areas and developing parts of the affected countries in West Africa which is the focus of our research. These impoverished areas we may categorize as low socio-economic status population. Socioeconomic status has been a powerful determinant of health since people in that group hardly can afford the cost of maintaining good health. There are many complex factors and pronounced invariability in the relationship between socioeconomic status and health. The more opportunity a group has in terms of education, social class and income, the more they tend to have better health. This is the more reasons why Ebola disease is very pronounced in most developing countries like African countries. As generally obtainable, rich and substantially well-to-do people tend to be in better health than people of poorer status because the rich ones can afford to live in serene and comfortably clean environment and equally afford medical expenses when the need arises, Didigwu *et al*. (2019).

Because most of the affected areas are in rural areas of developing countries where communication by roads and even networks are difficult, medical teams responding to the Ebola epidemic control in some West African and other developing countries were confronted with the problems of locating people living in distant isolated areas due to lack of development. Maps of these rural areas/locations in most cases do not exist, are not accurate, or are very obsolete. Fundamental information on location of houses, buildings, villages and roads were not easily obtainable, thereby making contact tracing and detection of affected areas severely difficult. The situation is worsened as a result of inadequate or lack of modern information and communication gadgets in these areas. Further complication of the matter is the local miss-belief about the disease, ignorance of implications of local culture and beliefs and lack of participation of local communities in the disease control efforts. As a result of the poor socioeconomic development of these remote areas and villages in developing countries, there is distrust between these rural communities and those seeking to control the epidemic and this to a large extent contribute to the unwillingness of the members of these communities to be involved and take part in the control effort. There have been reports of poor management of suspected victims admitted at the transit centers (TC), because it takes up to three days or more before the results of their tests are made available as a result of lack of transportation of the specimens to the urban areas for analysis. Furthermore, debilitated healthcare systems, mistrust of government after several years of armed conflict, and the delay in responding for so many months, all contribute to the failure to control the outbreak. Other factors included local burial customs of washing the body and the unprecedented spread of Ebola to densely populated cities.

Ebola virus has been identified in saliva, breast milk, stool and tears of acutely ill patients. Even when the symptoms of the Ebola disease were no longer detectable in a patient’s blood, the virus was still found present in breast milk. It was equally shown in some research works that the virus can be present in semen 40 days after the virus symptoms began (Ahman *et al*; 2021). For these reasons, people are encouraged to practice abstinence from both breastfeeding and unprotected sex immediately after recovering from Ebola virus disease (Bausch *et al.*, 2007), (Ahman *et al;* 2021). When infected with Ebola disease, the symptoms of this disease may appear as from between two to twenty-one days. If an individual has no symptoms, he will not be infectious. Researchers showed that the virus is not detectable in the blood before symptoms manifest.

On the issue of host to the virus, it is speculated that arthropods, rodents and bats are the host for Ebola virus (Chowell and Nishiura, 2014; Sullivan *et al.*, 2003). Direct transmission from reservoirs or secondary infected animals may occur. Bush animals may transfer the virus to humans when infected and human comes in contact with their infected and infectious body fluids or when their meat is eaten by humans uncooked or under-cooked. In this situation, the virus then spreads in the human population through human-to-human transmission. Airborne spread of the disease has not been detected or discovered in Ebola outbreaks, (Washington state department of health, 2018). A study carried out in December 2016 showed that the use of the vaccine of rVsv-EBOV is 70% to 100% effective in protecting persons against infection of the Ebola virus. According to Gimm and Nicholas (2015), two main strategies to stop the spread of Ebola are to diagnose infected people and people in danger of getting infected and prevent further disease spread by isolating symptomatic and infectious individuals. Furthermore, tracing the contacts of the asymptomatic infected ones should be carried out and such should be quarantined. Safe burials for those that died of the disease should be held since their bodies contain the virus and thus also infectious.

## Materials and Methods

Having discussed in detail the nature and process of spread of this virus in a developing country and noting the peculiar features of the areas in question, we now develop the mathematical model representing this information obtained that can enable us to carry out some analyses and provide vital information for the medical health officials about the disease caused by the virus. To do this successfully, we have our model assumptions as:

### Assumptions of the model equations

1. There is no vertical transmission of the infection from mother to unborn baby.
2. There is no asymptomatic infection so that for an individual to be able to infect others, the individual must be infectious.
3. There is homogeneous mixing meaning that all susceptible individuals have equal likelihood of becoming infected once in contact with infectious individuals.
4. There are intervention procedures namely, quarantine for the exposed, isolation for the infectious, treatment drug for both exposed and the infectious, vaccine drug for a portion of the susceptible population and condom usage by some agreed individuals whether vaccinated or not.
5. Isolated individuals are under close surveillance and do not contribute to the transmission of the infection as those treating them have proper and required preventive measures while the dead ones resulting from this disease in this class of persons are properly buried.
6. Disease induced deaths only take place in the infectious compartments while natural death rate is the same in all the compartments.
7. There is no herd immunity in the population.
8. There are available and accessible Vaccine, condoms, treatment drug, place of quarantine for the exposed and place for isolation for the infectious for all those that needed them.
9. The recovered individuals from the Ebola virus disease become susceptible to the virus again after some time.
10. There are immigration and birth into the susceptible population and there is no inherent immunity to the disease in the population.
11. The infectious compartment is a transition point so that the infected and infectious individuals do not stay long before joining either the isolated, treated or not treated compartments.
12. The materials and instruments used in the treatment of and caring for infectious persons such as syringes, beddings and clothes are properly disposed of and or buried.
13. The Ebola virus can still infect persons through semen from recovered individuals for some months after recovery
14. The recovered individuals from the disease can become susceptible to the virus again after some time.
15. The Ebola infected and infectious animals in the population have interactions with the susceptible animal and human populations.

With these assumptions, the total human and animal populations were compartmentalized based on the disease status of those involved. Thus we have: The human individuals that can readily contact the disease if in contact with an infectious person which we call the susceptible individuals, **S**: The susceptible portion of the population that are vaccinated, **S_v_**: The susceptible portion of the population that agreed not to be vaccinated called the unvaccinated individuals, **S_u_;** The susceptible vaccinated individuals using condoms, **S_vc_;** The susceptible vaccinated individuals not using condoms, **S_vn_;** The susceptible unvaccinated persons using condoms, **S_uc_**; The susceptible unvaccinated persons not using condoms, **S_un_**:, The individuals that have been infected by the disease but have not yet produced any symptoms which we call the exposed individuals, **E;** The exposed individuals that are taking treatment, **E_T_**, The exposed individuals that are quarantined, **E_Q_;** The infected and infectious individuals, **I**; The infected and infectious persons who are isolated while undergoing treatment, **I_i_**; The infected and infectious individuals that are normally treated but not isolated, **I_T_;** The infected and infectious individuals that are not treated, **I_N_**, The individuals that were treated and they recovered from the viral disease, **R;** The individuals that died of the viral disease and are yet to be buried but in contact with susceptible persons, **D_u_**, The animal population that can be infected by the virus and become infectious to other animals and human beings if in contact with infectious animals or persons, **S_r_**; The susceptible animals that are exposed to the virus, **E_r_**; The infected and infectious animals in the animal population, **I_r_**.

The mathematical model equations we obtained using the above assumptions and descriptions are


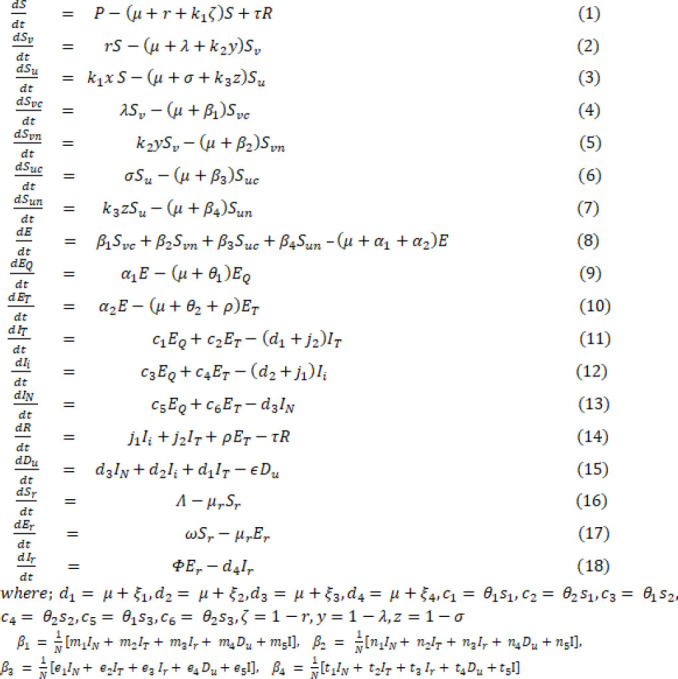


The parameters of these modelled equations are described as:

Ρ: The number of new recruitments into the susceptible human class.

Λ: The number of new recruitments into the susceptible animal class.

μ: The rate at which all human beings die due to natural death.

μ_r_: The rate at which all animals die due to natural death.

r: The proportion of the susceptible individuals that are vaccinated.

(1-r): The proportion of the susceptible individuals that are unvaccinated.

k_1_: The probability that the unvaccinated individuals remain unvaccinated.

λ: The rate at which the susceptible vaccinated individuals use condoms.

(1-λ): The rate at which the susceptible vaccinated individuals do not use condoms.

k_2_: The probability that the vaccinated individuals not using condoms remain not using

condoms.

σ: The rate at which the susceptible unvaccinated individuals use condoms.

(1-σ): The rate at which the susceptible unvaccinated individuals do not use condoms.

k_3_: The probability that the unvaccinated individuals not using condoms remain not using condoms.

β_1_: The rate at which susceptible vaccinated condom using individuals become exposed to the virus.

β_2_: The rate at which susceptible vaccinated not using condom individuals become exposed to the virus.

β_3_: The rate at which susceptible unvaccinated condom using individuals become exposed to the virus.

β_4_: The rate at which susceptible unvaccinated not condom using individuals become exposed to the virus.

α_1_: The proportion of the exposed individuals that are quarantined.

α_2_: The proportion of the exposed individuals that are undergoing treatment.

θ_1_: The rate at which the exposed and quarantined individuals become infectious.

θ_2_: The rate at which the exposed individuals undergoing treatment become infectious.

s_1_: The proportion of the infectious individuals that are undergoing treatment.

s_2_: The proportion of the infectious individuals that are isolated and undergoing treatment

s_3_: The proportion of the infectious individuals that do not get any kind of treatment.

ξ_1_: The rate at which the infectious individuals undergoing treatment die of the disease.

ξ_2_: The rate at which the infectious and isolated individuals undergoing treatment die of the disease.

ξ_3_: The rate at which the infectious and not treated individuals die of the disease.

ξ_4_: The rate at which the infectious animals die of the disease.

j_1_: The rate at which the infectious individuals undergoing treatment recovers from the disease.

j_2_: The rate at which the infectious and isolated individuals undergoing treatment recovers from the disease.

ρ: The rate at which the exposed individuals undergoing treatment recovers from the disease.

τ: The rate at which the recovered individuals become susceptible to the virus again.

ω: The rate at which the susceptible animals become exposed to the virus.

*ɛ*: The rate of proper burial for the dead and unburied population.

Φ: The rate at which the exposed animals become infectious.

for *n_1_, n_2_, n_3,_ n_4_* being the infective rates of S_vn_ in the infective compartments

*m_1_, m_2_, m_3_, m_4_* being the infective rates of S_vc_ in the infective compartments

*e_1_, e_2_, e_3_, e_4_* being the infective rates of S_uc_ in the infective compartments

*t_1_, t_2_, t_3_, t_4_* being the infective rates of S_un_ in the infective compartments

### Model Analysis

Having obtained the model equations, we need to analyse these equations to see if they will give a good representation of the disease transmission dynamics. But before this, we first authenticate the well-posedness of the model by showing that the model satisfied the conditions on the positivity of the variables of the model as well as the continual remaining of the variables in the region of existence of the variables. Thus, we have the theorem:

### Theorem

Let us consider the region defined as:


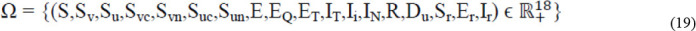


We prove that all solutions of the system (equations 1 - 18) are positive for all t > 0 provided that the initial conditions are positive. For the system of equations (1 - 18), the region Ω is positively invariant and the solutions starting in Ω stays in Ω.

### Proof

Suppose that the initial conditions on the variables are all positive, that is:


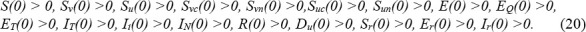


We then prove by contradiction, according to Bolarinn and Adeboye, (2011), Onah et al. 2020, that the solutions of the system of equation (1-18) are all negative. We assume for a contradiction that there exists t_1_ such that;


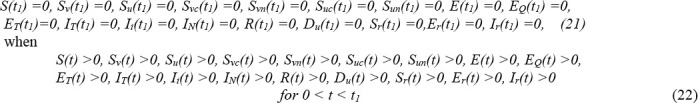


Now, if S(t_1_) = 0, then


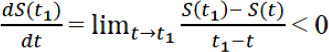


Similarly, if S_v_(t_1_) = 0, then we also get that


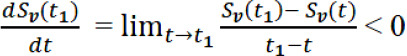


We can continue in the same way to prove it for the remaining variables.

Then from equations (1 – 18) and equations (21) and (22), 
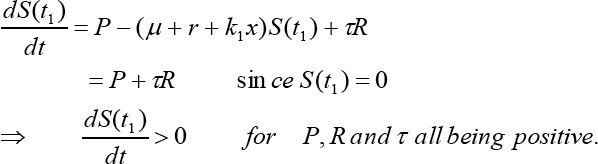


Thus, this contradicts our earlier assumption which implies that *Ѕ(t*_1_)≠ 0 and S will remain positive for all t. With similar arguments, we obtained that:


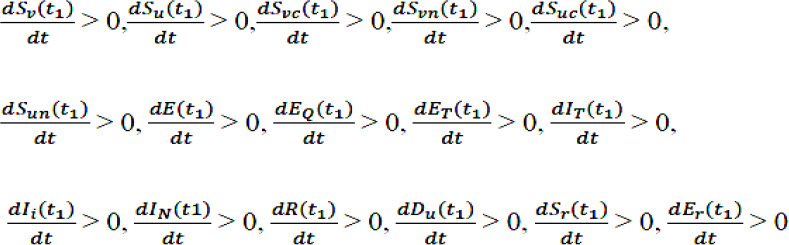


Thus, in general, we have that:


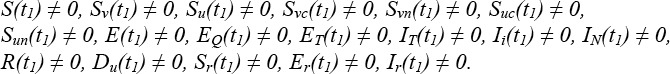


By this, we have shown that all the solutions of the equations in the system (equations 1 - 18) are positive and in ℜ_+_^18^, provided that the initial conditions are positive. Thus, Ω is positively invariant, which implies that the model is mathematically well posed and reasonable which can be used to study the transmission of the disease in question.

Our next point of analyses is the continuous existence and non-existence of this disease in the population. This is studied by analyzing to get the equilibrium points of the disease at the Disease free and the endemic states. At these points, it is expected that the virulence of the disease does not change. Thus, we study the equilibrium state as follows:

### Disease free equilibrium

Let *E (S, S_v_, S_u_, S_vc_, S_vn_, S_uc_, S_un_, E, E_Q_, E_T_, I_T_, I_i_, I_N_, R, D_u_, S_r_, E_r_, I_r_*) be the equilibrium point of the system (equations 1 - 18). At equilibrium state (Unaegbu, Onah and Oyesanya; 2021), we have that


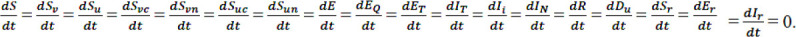


This then means that the right-hand sides of equations (1 – 18) will be equated to zero. Solving these resulting equations, we obtain the equilibrium point ε_0_ as:


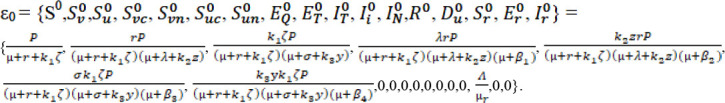


Thus, at the disease-free equilibrium, all the diseased compartments are zero whether in the human or the rodent population.

### The endemic disease equilibrium state

The endemic disease equilibrium state is the state where the disease cannot be totally absent in all the various compartments in the population. For the disease to persist in the population, the susceptible class or compartment, the vaccinated class, the exposed class, the infectious class, the recovered class, and others must not all be zero at this equilibrium state (Ahman *et al*; 2020a). In other to obtain the endemic equilibrium points of the model, we solve equations (1 – 18) simultaneously after we might have equated the left-hand-side of equations (1 – 18) to zero, that is,


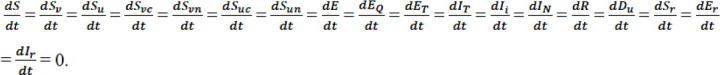


to obtain the following;


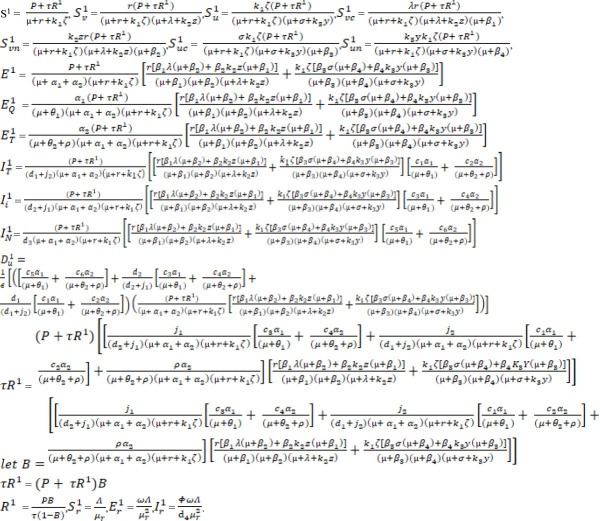


Thus, the endemic equilibrium points of the model equation (1 - 18) exists and is given as


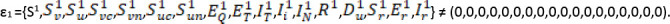


Having established these equilibrium points, we need to verify whether these equilibrium points are stable or not. Thus, we study the stability of the disease free and endemic states of this disease at their equilibrium points. We have two types of stability: the local and global stability of the disease states.

### Local stability

By local stability of the disease state, we mean a change in the disease state due to a small variation in the state of the disease level. If the state of the disease remains the same with the little variation in the disease level, we say that the disease is stable in the population (Onah *et al;* 2020). We have the local stability analysis in the disease free and endemic state. In the disease-free state, we have that β_1_ =0, β_2_ = 0, β_3_ = 0 and β_4_ = 0. We linearize the system of equations (1-18) to obtain the Jacobian matrix


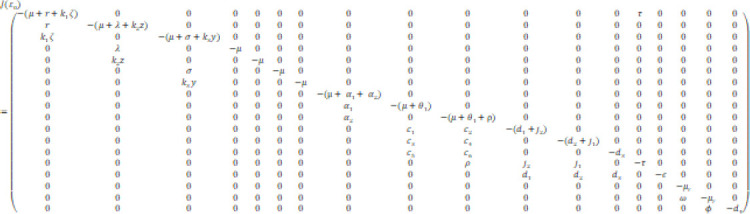


Evaluating the Jacobian matrix J(ε_0_) we have the following corresponding eigenvalues


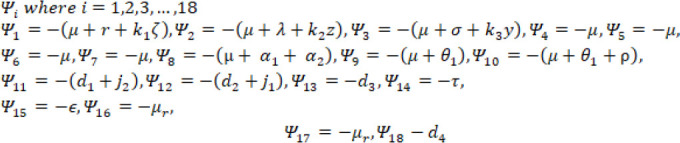


As can be seen, all the eigenvalues have negative real parts implying that the disease-free equilibrium state is locally asymptotically stable.

In the disease-endemic state, we have that β_1_ ≠ 0, β_2_ ≠ 0, β_3_ ≠ 0 and β_4_ ≠ 0.

We studied the local stability of the endemic equilibrium using the Centre Manifold theorem (Castillo-Chavez and Song; 2004), We define the equation as 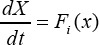 where F_i_(X) = (f_1,_ f_2,_ f_3…_ f_18_) (X)

Then we redefine the equations (1 - 18) as:


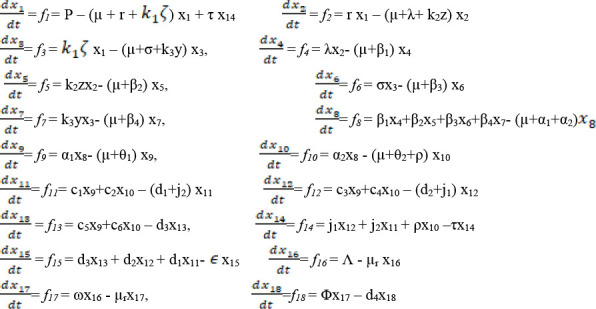


We applied the central manifold theorem and obtained the bifurcation coefficients a < 0 and b < 0 (Ahman *et al*; 2021), this shows that the endemic equilibrium state is locally asymptotically stable and at the same time exhibits forward bifurcation. Now we can study the global stability.

## Global stability of the equilibrium states

Global stability analysis is one of the classical approaches considered very vital in mathematical epidemiology. In this, we studied what happened at the equilibrium levels when the disease level is altered relatively not very small. If this is such that at very short interval of time, the diseased state returns to the former equilibrium state, then we say that the equilibrium state is globally stable and if otherwise, it is globally unstable. To carry out this study, we used a special technique called the Lyapunov function technique. This technique has been used by many authors in their works.

As in the former case, we studied the global stability of system (equations 1 - 18) for the disease-free equilibrium and then endemic equilibrium cases.

### Global stability of the disease-free equilibrium state

To carry out this study and referring to the works of Atokolo et al. (2020) and Ahman et al. (2021), we use the **LaSalle’s invariant principle** (Lassalle; 1976) to define a Lyapunov function:


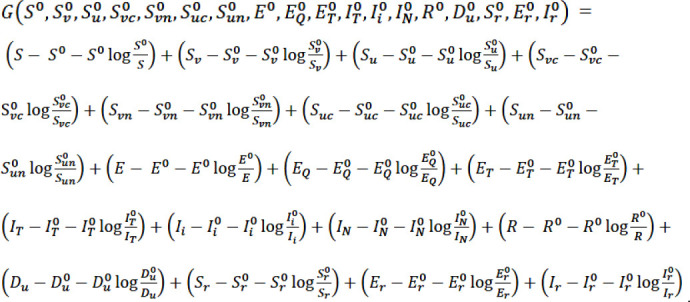


But at the disease-free equilibrium, 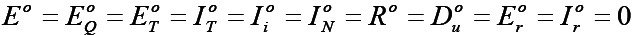 so that evaluating the derivative of G, we obtain:


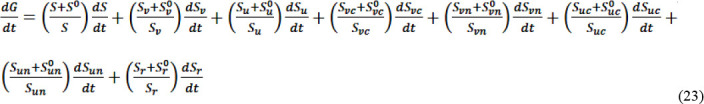


Substituting for the derivatives in equation (23) above using equations (1 – 18) and simplifying, we then obtain:


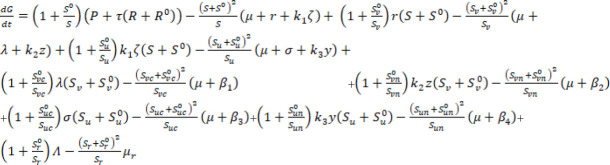


If we collect the positive and negative terms in the above equation, we then have


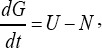


Where,


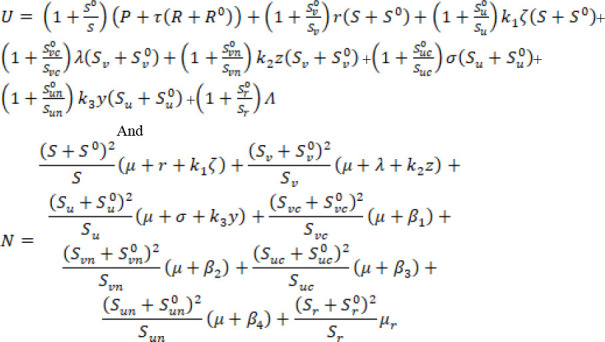


When 
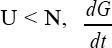
 will be negative definite along the solution path of the system. This implies that 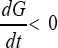 and 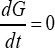 only at the disease-free equilibrium ε_o_. This according to LaSalle’s invariant principle therefore implies that the disease-free equilibrium is globally asymptomatically stable in Ω.

### Global stability of the endemic equilibrium state

To obtain the global stability of the endemic equilibrium state of the disease in the population, we adopt the same method as above but now using the values of the variables at the equilibrium state.

We consider the following Lyapunov function stated as:


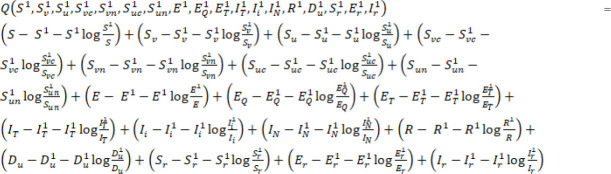


But at the disease-free equilibrium, 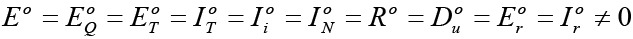


Carrying out the same method of simplification, we obtain:


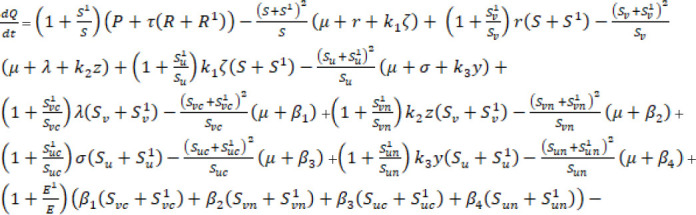



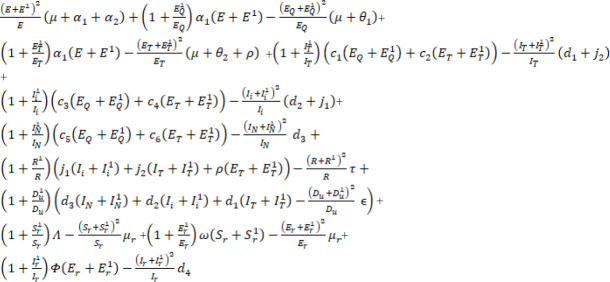


Collecting the positive and negative terms we have the following expression;


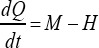


Where:


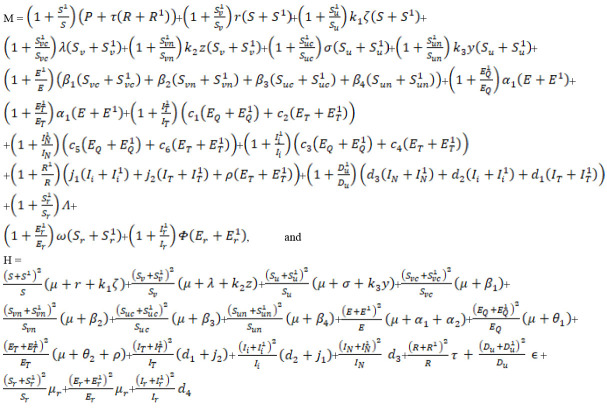


When M < H, then 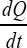 will be negative definite along the solution path of the system which implies that 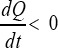 while 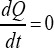 at the disease-endemic equilibrium ε_1_. This according to LaSalle’s invariant principle therefore implies that the endemic equilibrium is globally asymptomatically stable in Ω.

## Results

### Numerical Simulations

Numerical simulations of the system (equations 1 – 18) are carried out to further investigate the transmission dynamics of Ebola virus disease and to complement the mathematical analysis we carried out in the previous sections. We thus apply this Ebola virus disease model on a West African country called Liberia which according to World Health Organization, the spread of Ebola virus disease officially started there on the 16th of June 2014 with 33 cases and 24 deaths, Althaus (2014). The population of Liberia was estimated to be 4,396,554 in 2014 (WHO; 2016a). Then we estimated our initial conditions as S(0) = 4396554 – 33 = 4,396,521, I(0) = 33, D_u_(0) = 24, E_Q_(0) = 107 -33 = 74. This is because, 107 is the number of confirmed cases as at 30th of June 2014, Madubueze et al. (2018). Also, E (0) = 0, E_T_ (0) = 0, I_i_ (0) = 33 – 24 =9, I_T_ (0) = 0, I_N_ (0) = 0,

R (0) = 0.

A phase-3 ring vaccination cluster- randomized trial reports the efficacy of the vaccine in different scenarios. In individuals who randomly received the ring vaccination, the vaccine was 100% efficacious after 10 days which requires 42.2% - 63.0% of the population to be vaccinated to provide herd immunity in the population. Since we assumed there is no herd immunity in the population, we then estimate 40% of the population to be vaccinated and 60% unvaccinated. Thus, S_v_ (0) = 1,758,608, S_u_ (0) = 2,637,913, S_vc_ = 527,582,

S_vn_ (0) = 1231026, S_uc_(0) = 1,582,748, S_un_(0) = 1,055,165.

Assuming the total population of animals that can be infected with Ebola virus disease and can infect human beings with the virus that is in Liberia is 6,000. Then with 6000 susceptible animals at the initial time, we have S_r_ (0) = 5680, E_r_ (0) = 120, I_r_ (0) =200.

## Results and Discussions

The following are the results of the model simulations using our initial conditions and parameter values in Table 1 with MATLAB software:

**Table 1:** Model Parameter Values

Parameter	Values	Source	Parameter	Values	Source
P	422	Madubueze *et al.* (2018)	ω	0.5	Assumed
Μ	0.00004	Assumed	τ	0.06	Rivers *et al.* (2014)
μ_r_	0.08	Assumed	Φ	0.6	Assumed
R	0.4	Bhunu,(2015)	ρ	0.0314862	Rivers *et al.* (2014)
Λ	0.03	Assumed	α_1_	0.07143	Gomes *et al.* (2014)
Σ	0.07	Assumed	α_2_	0.41	Assumed
Λ	100	Assumed	θ_1_	0.08333	Legrand *et al.* (2007)
s_1_	0.2257	Gomes *et al.* (2014)	θ_2_	0.014	Assumed
s_2_	0.25	WHO (2014)	ξ_1_	0.11386	Gomes *et al.* (2014)
s_3_	0.148	Assumed	ξ_2_	0.0901	Assumed
k_1_	0.895	Assumed	ξ_3_	0.3110	Assumed
k_2_	0.04	Assumed	j_1_	0.05186	Gomes *et al.* (2014)
k_3_	0.02	Assumed	j_2_	0.17	Rivers *et al.* (2014)
β_1_	0.04	Estimated	n_1,_n_2,_n_3,_n_4_	0.0001-0.0005	Assumed
β_2_	0.1	Estimated	m_1,_m_2,_m_3,_m_4_	0.0001-0.0005	Assumed
β_3_	0.5	Estimated	t_1,_t_2,_t_3,_t_4_	0.0001-0.0005	Assumed
β_4_	0.3	Estimated	e_1,_e_2,_e_3,_e_4_	0.0001-0.0005	Assumed
*ɛ*	[0 - 1]	Assumed			

**Figure 1:**
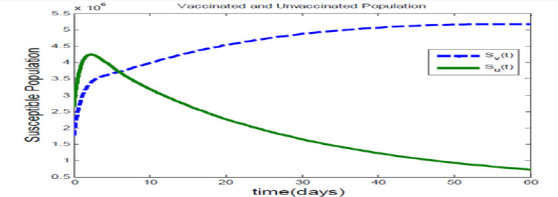
Comparison of Susceptible vaccinated and Susceptible Unvaccinated populations

Figure 1 above shows the two susceptible human populations: the susceptible vaccinated and the susceptible unvaccinated populations. Given that the number of people being vaccinated is ever going on, we can see that if vaccination is continued for a long time, almost every member of the population will be vaccinated leading to reduction in the number of the unvaccinated. However, within the first ten days of starting the investigation with the vaccination going on, the total population of the unvaccinated is higher than the vaccinated population. The importance of this graph is that it shows that if vaccination is a good control strategy against the disease, as found in the case of this disease, it will be advisable to enforce vaccination, and this should be done for a relatively long period of time to cover a relatively large proportion of the susceptible population. This in effect will then reduce the infectivity of the disease in the population.

**Figure 2:**
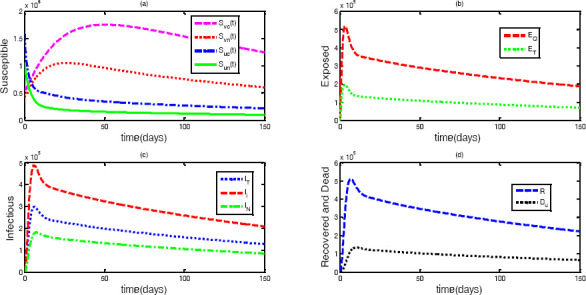
Graph of the model Compartments

**(a)** shows the populations of the susceptible vaccinated and unvaccinated compartments that are either using or not using condoms. Form the figure, we can see that the lowest susceptible population is that of the unvaccinated group that are not using condoms, (S_un_). This is because, this group or compartment do not have any kind of protection against the infection. They had neither vaccination nor even condom usage while engaging in sexual activities. As expected, the second lowest is still the susceptible unvaccinated but using condom while engaging in sexual activities. At least, this group has one form of protection against infection. We can equally see that the best option to retain large population still free of the disease is to vaccinate the population and enforce that usage of condom while engaging in sexual activities must be observed. Therefore, for proper control of this deadly disease in developing countries, a lot of energy and money must be spent in making the populace to know (Enlightenment campaign) the importance of vaccination and usage of condom when engaging in sexual activities. Most importantly, the condom and vaccine must be made readily available and at affordable cost to the most vulnerable groups. Furthermore, the negative myth and or interpretation of vaccination must be strongly discountenanced as many do believe that vaccination is a strategy by the whites (Western world) to reduce the population of the people in developing countries.

**(b)** In this figure, we can see that populations in either of the two compartments take the same shape even though at differing levels. As expected, we can see that the population of those under quarantine is larger than those for treatment. This is because, those whose clear status are not known but might agree to go for treatment must be those who are very sure that they had direct contact or interaction with an infectious person. All other persons who are in doubt of their status readily go for quarantine which in reality constitutes that larger population of the exposed.

We can see that the peak of the exposure period is small but the exposed class remains as far as there are interactions between the susceptible and infectious persons in the entire population.

**(c)** In this figure, we have three types of infectious compartments: the infectious ordinarily undergoing treatment in the main ward, the infectious undergoing treatment but isolated and then the infectious not undergoing any treatment. One would expect that those infectious and not undergoing any form of treatment will constitute the bulk of the infectious population. This is not so because the fatality rate of this disease is high such that once infectious and not receiving any form of treatment, the person will die. It is also expected that the population of those infectious but being treated in open wards should be next because; proper attention is not given to them as does for the isolated persons. Furthermore, because of the infectiousness of the disease and the fatality rate, most infectious persons are treated in total isolation to ensure survival and reduce infecting other people. Due to none or hardly any existence of isolated units in hospitals and health institutions in most developing countries, the fatality rate of this disease and equally the rate of generation of new infection is very high. This therefore goes to show that isolated treatment of infectious patients must be encouraged in developing countries by providing the necessary facilities in the hospitals and health institutions.

**(d)** In this figure we show the populations of the recovered persons as well as those that are dead and unburied. When treatment is administered, many infectious persons recover but still a very good number of them die. Note that there are some infectious persons who never went for any treatment. Most people in this group are usally from the rural areas where most of the time, the usual traditional burial processes are observed. In this case, proper preventive measures to avoid infections from this infectious dead persons are not usually observed in such burials. We call such dead persons as unburied because within this period of observation of this burial processes, the dead body is infectious and really infecting very many persons due to contact either during bathing the corpses or dressing the corpses. In other to reduce the transmission level of the disease in the community, all dead bodies due to the disease must be properly buried by the health officials duely observing all the preventive burial procedures. 

**Figure 3:**
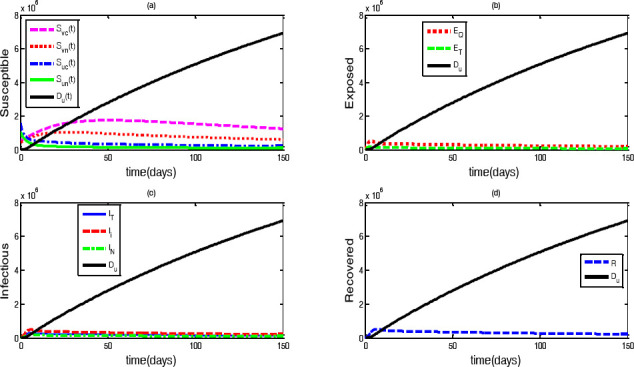
Gra ph of the Relationship between the Dead Unburied and other Compartments at 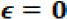

**Figure 3: (a) – (d)** shows that for each of the human population, when the rate of proper dead and unburied burial 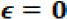 (i.e., when there is no proper burial of the dead unburied), the dead and unburied population will become high and keep increasing which will affect all other human population negatively. This means that when the diseased dead and unburied (which are infectious) in the population is not buried properly at all, it will drastically infect and affect the entire population. This outcome can be avoided by putting in place a proper burial system that will help in making sure that all the diseased dead unburied persons are properly buried. 

**Figure 4:**
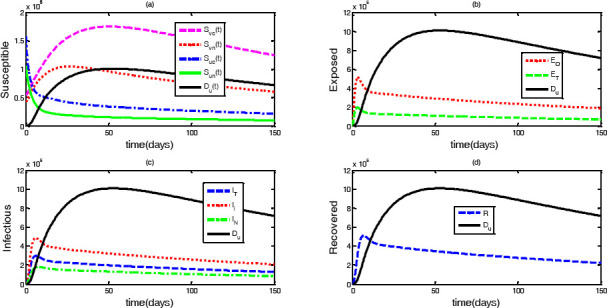
Graph of the model and relationship between the Unburied and other Compartments at 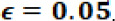

**Figure 4: (a) – (d)** shows for each of the human population the effect of the rate of proper burial of the dead and unburied infectious burial at 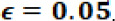 (i.e., when there is little proper burial of the dead and unburied infectious persons). Here is shown that the dead and unburied infectious population will initially increase and later decrease which will still affect all other human populations negatively because it is contributing to the disease spread. This outcome can also be avoided by putting in place a proper burial system that will help in making sure that all and not few of the diseased dead and unburied persons are properly buried.

**Figure 5:**
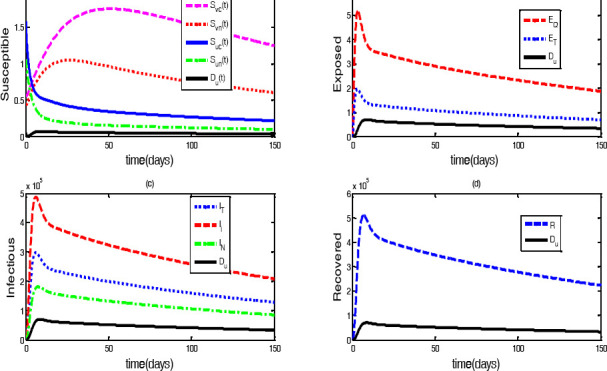
Graph of the model and relationship between the Unburied and other Compartments a 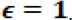

**Figure 5: (a) – (d)** shows for each of the human population the effect of the rate of complete proper burial for the dead and unburied infectious persons, which is at 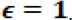 (i.e., when there is absolute proper burial of the dead and unburied infectious persons). From here, we can see that the dead and unburied infectious population is very low and thus will give a better control of the disease in the population This outcome showed that no matter other control measures applied, it is proper and very important to include the control measure of proper burials for the dead and unburied infectious persons which has been shown to contribute immensely to control of the disease.

## Conclusion

In our study here, we were able to formulate an elaborate mathematical model that contain majority of the features obtainable in developing countries that influences the wide and quick spread of Ebola Virus disease in the population. We included the issue of washing of dead bodies by the villagers thereby making the dead bodies one of the major sources of contacting and spreading the disease. We equally introduced the idea of Vaccination, treatment, quarantine, isolation while treating infectious persons and the use of condoms as obtained in developed countries to see their impacts in controlling the disease in developing countries.

In the analyses of our modeled equations, we obtained the equilibrium points of the transmission of the disease and further analyzed these equilibrium points to obtain the conditions for the local and global stabilities of the disease free and endemic equilibriums of the disease transmission dynamics. Stability analysis explains the impact of introduction of new infectious persons into the already infected and disease rampaging population. If introduction of few infectious persons into the population does not cause large or meaningful increase in the endemicity of the disease in the population, we then say the disease spread and level in the population is locally asymptotically stable and unstable if otherwise. If on the other hand, large inflow of infectious persons into the population does not cause any noticeable change in the diseased state of the population, then we say that the transmission of the disease in the population is globally stable.

It can be seen in our graphs that isolation is the best option for an infectious person to be treated so that he may not spread the disease further and can recover better. We equally studied through the simulation the impact of the vaccination with and without using condoms which showed that vaccine with condom use is the best control option for the human susceptible population. Also, from our graphs the proper burial control of the diseased, dead and unburied persons in the population has a great effect on the disease spread and control.

In our further publications, we are going to carry out sensitivity analyses to see the effect of the control, transmission and treatment parameters on the spread of the disease in the population. We shall further carry out study on the impact of the socio-economic classes in the spread of the Ebola Disease in the developing countries of the world.

### Declaration of Conflict of Interests

The authors declare that they have no competing interest associated with this study.

**List of Abbreviations:**
(EBOV), Zaire Ebola virus
(SUDV), Sudan Ebola virus
(BDBV), Bundibugyo Ebola virus
(RESTV): Reston Ebola virus
(TAFV), and Tai Forest (Cote D’ivore) Ebola virus
(EVD), Ebola virus disease
(CDC), Center for disease control
(TC): Transit Centers
